# Regular, narrow QRS, long RP tachycardia – what is the mechanism?

**DOI:** 10.1007/s12471-016-0897-4

**Published:** 2016-09-16

**Authors:** S. Tzeis, S. Pastromas, A. Sikiotis, G. Andrikopoulos

**Affiliations:** Pacing and Electrophysiology Department, Henry Dunant Hospital Center, Athens, Greece

## Answer

The differential diagnosis of a regular, narrow QRS, long-RP tachycardia includes atypical atrioventricular nodal reentry tachycardia (AVNRT), atrial tachycardia and atrioventricular reentry tachycardia (AVRT) via a slowly conducting accessory pathway usually presenting decremental conduction properties.

Ventricular overdrive pacing is the proposed initial diagnostic manoeuvre. During ventricular overdrive pacing the following criteria are assessed: (A) post-pacing response (V-A-V versus V‑A-A-V) and (B) post-pacing interval (PPI) minus tachycardia cycle length (TCL). A V-A-A-V response strongly suggests atrial tachycardia, while a V-A-V response is encountered in both AVRT and AVNRT [1]. The PPI-TCL differentiates an atypical AVNRT from an AVRT with a discriminant value of 115 msec (>115 msec suggests atypical AVNRT, while <115 msec an AVRT) [2].

**Fig. 1:**
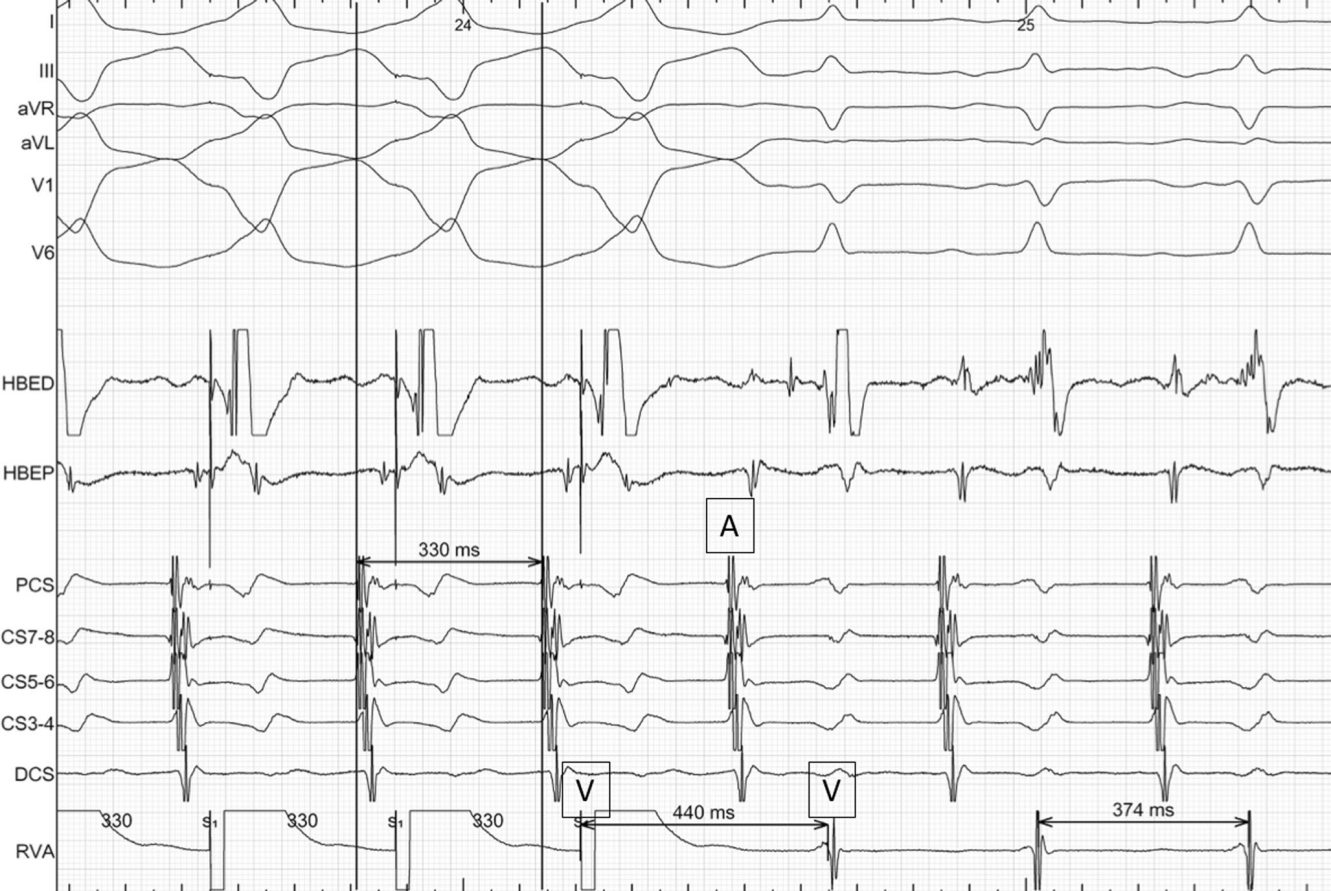
Ventricular overdrive pacing from the RV apex with retrograde atrial capture during the regular, long-RP, narrow QRS tachycardia (cycle length 370 ms). The post-pacing response demonstrated a V-A-V pattern with a PPI-TCL <115 msec which leads to the diagnosis of an atrioventricular reentry tachycardia using a slowly conducting accessory pathway. From top to bottom surface ECG leads (I, III, aVR, aVL, V1 and V6) and electrograms recorded from the distal and proximal bipole of a catheter located at the His (HBED: distal bipole, HBEP: proximal bipole) and a decapolar catheter placed in the coronary sinus (PCS: proximal coronary sinus, DCS: distal coronary sinus)

**Fig. 2:**
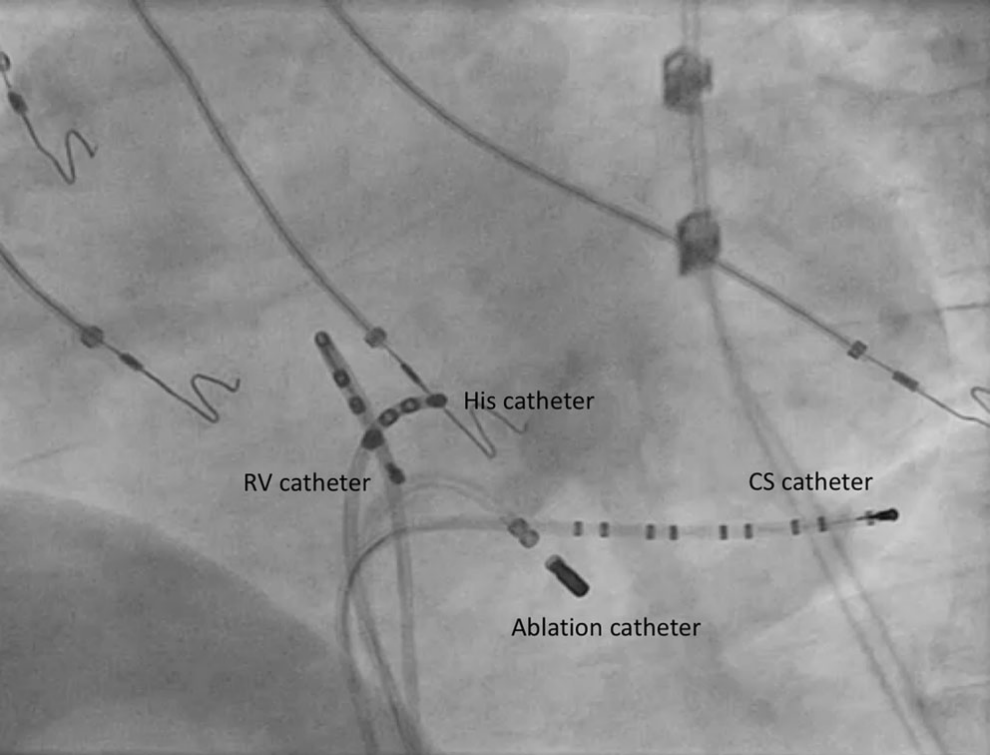
Fluoroscopic image of the site of successful ablation of the accessory pathway as shown in a left anterior oblique projection. The course of the trunk of the coronary sinus is demarcated by the decapolar catheter while the ablation catheter is located in a posterior branch of the coronary sinus

In our case, ventricular overdrive pacing resulted in consistent retrograde atrial capture, V‑A-V post-pacing response, with a PPI-TCL of 64 msec which led to the diagnosis of an AVRT (Fig. 1). During mapping, the earliest retrograde atrial activation was identified in a coronary sinus branch, suggestive of a coronary sinus-ventricular accessory pathway (Fig. 2) [3]. Ablation in the area of retrograde atrial prematurity with an irrigating catheter (20 W) resulted in tachycardia termination. Successful ablation was validated by post-ablation para-Hisian pacing, which showed a nodal response and adenosine administration during ventricular pacing.

The diagnosis of an AVRT using a slowly conducting accessory pathway should be taken into consideration in the differential diagnosis of a regular, narrow QRS, long-RP tachycardia even among patients with a first presentation within middle adulthood and an episodic occurrence on Holter recording, suggestive of an atrial tachycardia.
